# Gonadal tumor risk in pediatric and adolescent phenotypic females with disorders of sex development and Y chromosomal constitution with different genetic etiologies

**DOI:** 10.3389/fped.2022.856128

**Published:** 2022-07-22

**Authors:** Liangsheng Lu, Feihong Luo, Xiang Wang

**Affiliations:** ^1^Division of Pediatric Urology, Children’s Hospital of Fudan University, Shanghai, China; ^2^Department of Pediatric Endocrinology and Inherited Metabolic Diseases, Children’s Hospital of Fudan University, Shanghai, China

**Keywords:** gonadal tumor, Y chromosome, phenotypic female, children, gonadectomy, gonadal biopsy, disorders of sex development

## Abstract

**Objectives:**

This retrospective study sought to investigate the risk and proportion of gonadal neoplasms in phenotypic female pediatric patients with DSD and the presence of the Y chromosome and different genetic backgrounds in a single Chinese center.

**Materials and Methods:**

From January 2012 to December 2020, pediatric and adolescent patients with DSD and the presence of the Y chromosome who had unambiguous female genitalia and underwent bilateral gonadectomy or gonadal biopsy were included in this study. Patients’ demographics, karyotype, laboratory test results, gross pathology, and histology of gonadal tissue were all collected. The patients were divided into three groups based on their different genetic backgrounds, and the percentage of gonadal tumors was calculated to assess the risk of gonadal tumor and malignancy by etiology.

**Results:**

A total of 22 patients with DSD and an unambiguous female phenotype with a Y chromosome were recruited. The mean age was 10.91 ± 4.99 years (9 months to 19 years). Gonadal neoplasia was confirmed in six (27.3%) cases by pathological examination of surgical gonadal tissue samples. Among 44 gonadal samples from these 22 patients, the following were identified: five gonadoblastomas, three dysgerminomas, and two Leydig cell tumors. The youngest patient with a tumor was a 2-year-old girl with 46,XY complete gonadal dysgenesis (46,XY CGD or Swyer syndrome) and bilateral gonadoblastoma. Patients with 46,XY complete gonadal dysgenesis (4/6; 66.7%) had the highest tumor occurrence rate. Among 10 patients with Turner syndrome with the presence of the Y chromosome, only one patient was diagnosed with a gonadal tumor. Leydig cell tumor was diagnosed in only one of six patients with 46,XY androgen synthesis/action disorders.

**Conclusion:**

Pediatric patients with 46,XY complete gonadal dysgenesis had a significantly increased risk of developing gonadal tumors and underwent prophylactic gonadectomy as soon as the diagnosis was confirmed, whereas those with Turner syndrome with Y chromosome and 46,XY androgen synthesis/action disorders had a relatively low risk. In view of the limited number of patients, a large multicenter study with close follow-ups is needed to support these conclusions.

## Introduction

Patients with disorders of sex development (DSD), specifically disorders of gonadal formation and maintenance, are at a significantly higher risk of the development of gonadal tumors than the general population. Among the risk factors of gonadal neoplasia, the presence of a specific genetic locus, the so-called gonadoblastoma region on the Y chromosome (GBY region), is an important factor in the context of tumorigenesis in patients with DSD. Histological evaluation of gonadal tissue of patients with DSD will contribute to the identification of individuals with gonadal tumors. Gonadoblastoma (GB) and germ cell neoplasia *in situ* (GCNIS) are the two types of precursor lesions of gonadal germ cell cancer (GCC) in the context of DSDs. Approximately 50% of gonads containing GB are associated with infiltrating GCC components, and nearly all GCNIS eventually progress to GCC. Histological evaluation of gonadal tissue in patients with DSD is crucial for the identification of individuals with gonadal tumors. The highly sensitive and specific diagnostic markers like OCT3/4 and TSPY have been used as useful tools for the detection of GCNIS/GB and progression risk (1). Due to phenotypic and genotypic diversity, evaluation of the risk of development of gonadal tumors in patients with DSD is challenging. Hughes et al. (2) recommended prophylactic gonadectomy in females with bilateral streak gonads and the presence of Y-chromosome material. Few data are available on the risk of gonadal malignancy in patients with DSD with female external genitalia, who are typically diagnosed with DSD at the time of gonadal tumor diagnosis (3). Although significant research has been conducted to improve knowledge about the risk of gonadal tumors, a limited number of studies have focused on the timing and indications of gonadectomy during childhood and adolescence in female patients with Y chromosome abnormalities (4–8).

The optimal management of DSD in phenotypic females is especially controversial in the pediatric group, with differing views on the indications and timing for prophylactic gonadectomy (5, 7, 8). This single-center study reviewed the data of pediatric and adolescent phenotypic female patients with DSD harboring Y chromosomal material. The pathological findings of the removed gonads were analyzed to provide preliminary evidence for the risk and type of gonadal tumors in this rare pediatric group of patients. A brief literature review is included in the section “Discussion.”

## Patients and methods

### Patients and clinical data

**FIGURE 1:**
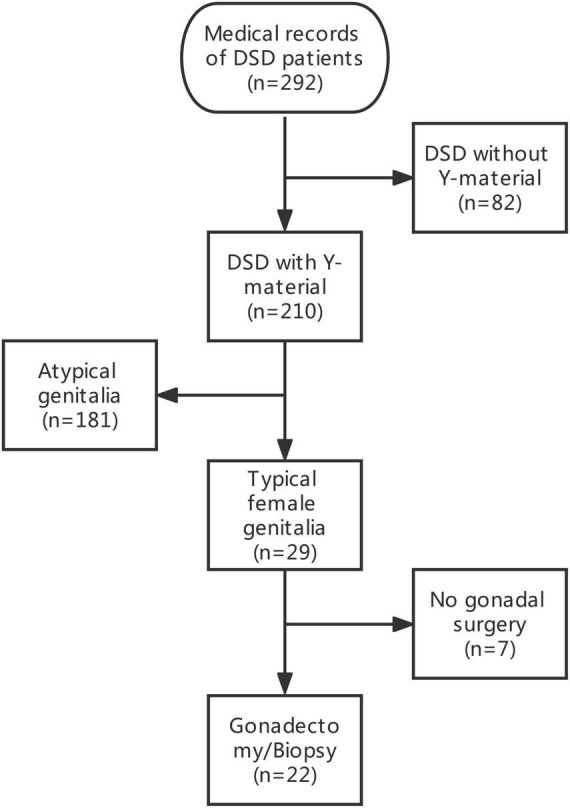
Flow diagram of section of patients with DSD according to the inclusion criteria.

The medical records of 292 patients with DSD in the Children’s Hospital of Fudan University between January 2012 and December 2020 were retrospectively reviewed. After excluding 82 patients with DSD without Y-chromosome material, 29 phenotypic female patients with DSD and unambiguous genitalia and with Y-chromosome material were identified among 210 patients with DSD and Y-chromosome material. The inclusion criteria included (1) DSD with the presence of the Y chromosome; (2) confirmed female external genitalia upon external genitourinary examination; and (3) underwent bilateral gonadectomy or gonadal biopsy. A total of seven patients were excluded because they did not undergo gonadectomy or gonadal biopsy. Therefore, a total of 22 patients were included in this study. The data of the selected patients were collected and included in this study (Figure 1). Demographics, karyotype, gross pathology and histology of gonadal tissue, ultrasound findings, laboratory test results, human chorionic gonadotropin stimulation, bone age determination, genetic tests, serum follicle-stimulating hormone (FSH) level, luteinizing hormone (LH) level, and testosterone level were collected (Tables 1, 2).

**TABLE 1:** Clinical presentations, hormonal profile, and karyotypes of the 22 phenotypic female patients with DSD.

	Age	Karyotype	Symptom	FSH^a^	LH^a^	*T* ^b^	E2^c^
1	10 years	46,XY	17OHD family history	N/A	N/A	N/A	N/A
2	15 years	46,XY	Amenorrhea	45.6	25.4	Nil	14
3	15 years	46,XY	Amenorrhea	30.8	147.4	449.9	35
4	14 months	46,XY	Bil IH	4.2	7.9	56.2	54
5	9 months	46,XY	Bil IH	2.4	6.2	109	71
6	9 years	46,XY	Short stature	31.5	0.8	Nil	Nil
7	2 years	46,XY	Chr abnormalities	42.3	2.4	Nil	20
8	14 years	46,XY	Amenorrhea	85.5	31.3	16	<5
9	10 years	46,XY	Abd mass	N/A	N/A	N/A	N/A
10	15 years	46,XY	Amenorrhea	38.9	11.3	27.9	25.9
11	15 years	46,XY	Amenorrhea	>200	230.9	48.1	<20
12	12 years	46,XY	Short stature	136.4	33.1	0.9	Nil
13	5 years	mos 45,X[8]/46,XY[12]	Short stature	N/A	N/A	N/A	N/A
14	10 years	mos 45,X[24]/46,XY[6]	Short stature	86.5	8.3	Nil	Nil
15	9 years	mos 45,X[5]/46,XY[45]	Short stature	11.7	0.3	Nil	1
16	19 years	mos 45,X[7]/46,XY[23]	Short stature	66.4	21.4	25.5	31
17	13 years	mos 45,X/47,XYY/46,XY	Short stature	72.7	12.9	<0.1	<20
18	14 years	mos 45,X/46,XY	Short stature	203	44.1	30	Nil
19	8 years	mos 45,X[11]/46,XY[59]	Short stature	N/A	N/A	N/A	N/A
20	14 years	mos 45,X[30]/46,XY[70]	Short stature	106.1	34.3	1.7	25
21	14 years	mos 45,X[4]/46,XY[46]	Short stature	143	22.4	26.8	26.2
22	15 years	mos 45,X/46,XY	Amenorrhea	82.2	17	18.4	10.9

^a^mIU/ml. ^b^ng/dl. ^c^pg/ml. Abd, abdominal; Bil, bilateral; Chr, chromosomal; IH, inguinal hernia; N/A, not applicable.

**TABLE 2:** Genetic abnormalities, procedures, pathological results, and diagnoses of the gonadal samples.

	Genetic abnormality		Procedure	Gonadal characteristics	Tumor	Diagnosis
	Gene	Genomic variation				
1	*CYP17A1*	c.1226C > G^a^ (11), c.707T > G^a^ (12)	LPBG	Bil testis	No	17OHD
2	*CYP17A1*	c.985_987delTACinsAA^a^ (13)	LPBG	Bil testis	Bil LCT	17OHD
3	*AR*	c.2245G > A^b^	LPBG	Bil testis	No	CAIS
4	*AR*	c.1314_1315del^b^	Biopsy + IHR	Bil testis	No	CAIS
5	*SRD5A2*	c.419_421delinsATTC^b^ c.547-1G > C^b^	Biopsy + IHR	Bil testis	No	SRD
6	*NR5A1*	c.244 + 1G > C (splicing)^b^	Biopsy	Bil testis	No	NR5A1
7	*SRY*+; WES(−)		LPBG	Bil streak gonad	Bil GB	Swyer Syn/TAPVD
8	*SRY*+; WES(−)		LPBG	Unil streak gonad; Unil tumor	Bil DG	Swyer Syn
9	*SRY*+; No further gene detection		Laparotomy + Tumor resection	Unil streak gonad; Unil tumor	Rt DG	Swyer Syn
10	*SRY*+; WES(−)		LPBG	Bil streak gonad	No	Swyer Syn
11	*SRY*+; No further gene detection		LPBG	Bil streak gonad	No	Swyer Syn
12	*SRY*+; WES(−)		LPBG	Bil streak gonad	Rt GB	Swyer Syn
13	*SRY*+; WES(−)		LPBG	Bil streak gonad	Bil GB	TS, SRY+
14	*SRY*+; WES(−)		LPBG	Bil streak gonad	No	TS, SRY+
15	*SRY*+; WES(−)		LPBG	Bil streak gonad	No	TS, SRY+
16	*SRY*+; No further gene detection		LPBG	Bil streak gonad	No	TS, SRY+
17	*SRY*+; No further gene detection		LPBG	Bil streak gonad	No	TS, SRY+
18	*SRY*+; No further gene detection		LPBG	Bil streak gonad	No	TS, SRY+
19	*SRY*+; No further gene detection		LPBG	Bil streak gonad	No	TS, SRY+
20	*SRY*+; No further gene detection		LPBG	Bil streak gonad	No	TS, SRY+
21	*SRY*+; WES(−)		LPBG	Bil streak gonad	No	TS, SRY+
22	*SRY*+; WES(−)		LPBG	Bil streak gonad	No	TS, SRY+

Bil, bilateral; Unil, unilateral; DG, dysgerminoma; GB, gonadoblastoma; IHR, inguinal hernia repair; LCT, Leydig cell tumor; LPBG, laparoscopic prophylactic bilateral gonadectomy; Rt, right; TAPVD, total anomalous pulmonary venous drainage. ^a^Variants reported before. ^b^Novel variations.

Based on chromosomal analysis, gonadal characteristics, and molecular diagnosis, patients were characterized as having one of the following etiologies: 46,XY androgen synthesis/action disorders, 46,XY complete gonadal dysgenesis (46,XY CGD or Swyer Syndrome), or Turner syndrome (TS) with a positive SRY gene (SRY+). The presence or absence of gonadal tumors was confirmed by pathological examination with immunohistochemical testing among 44 gonadal samples from these 22 patients. The risk of gonadal tumor was assessed for each etiology. This study was approved by the Ethical Committee of Children’s Hospital, Fudan University (approval no. 2022123). Informed consent for invasive procedures was obtained from the parents or legal guardians of the patients.

### Chromosomal analysis, gene detection, and molecular diagnosis

The karyotypes were generated from peripheral blood lymphocyte cultures, and the cytogenomic analysis was performed using G-banding. Abnormalities of the karyotypes were presented based on the International System for Human Cytogenomic Nomenclature (ISCN 2020) criteria (9). In this study, a comprehensive gene detection strategy was used for the molecular diagnosis of this group of patients with DSD and the presence of the Y chromosome. For patients suspected of having androgen insensitivity syndrome (AIS) and steroid 5α-reductase 2 deficiency after preliminary clinical assessment, targeted sequencing of *AR*, *SRD5A2*, and *SRY* exons was performed to detect variants by Sanger sequencing of PCR amplicons generated by custom-synthesized oligonucleotide primers. PCR amplification of the entire coding region of genes was done using the primers as described in our previous study (10). Recently, the advent and popularization of whole-exome sequencing technology has provided a well-justified strategy for finding candidate causative genes underlying genetic disease phenotypes. To identify the possible disease-causing mutations or variants in patients with unknown etiology and various DSD-related syndromes, we performed whole-exome sequencing (WES) to detect variants in the entire exon region of 20,000 genes in the human genome, on the basis of routine Sanger sequencing of *SRY* gene. The whole-exome sequencing was performed by NovaSeq 6000 series sequencers (Illumina, Inc., San Diego, CA, United States) using a whole exon capture chip (IDT The xGen Exome Research Panel V2.0, Integrated DNA Technologies, Inc., Coralville, IA, United States) with more than 99% coverage. All WES-detected variants suspected of pathogenicity were verified using Sanger sequencing by an ABI3730 sequencer (Thermo Fisher Scientific Inc., Waltham, MA, United States).

### Pathological examination of the gonadal samples

All surgical specimens were fixed in 10% neutral buffered formalin, embedded in paraffin, sliced conventionally, and stained with hematoxylin and eosin. The morphological changes were assessed, and the gonadal tumors were identified by macroscopic or microscopic examination with immunohistochemical confirmation by the detection of OCT3/4, PLAP, TSPY, and FOXL2.

### Statistical analysis

Statistical analyses included descriptive statistics to summarize demographic characteristics, karyotype, serum levels of sex hormones, and genetic test results. The risk of gonadal tumors was calculated using the percentage of patients with gonadal tumors. Means with standard deviations were reported as appropriate.

## Results

A total of 22 phenotypic female children and adolescent patients with DSD and the presence of the Y chromosome met the inclusion criteria; age at gonadectomy or gonadal biopsy ranged from 9 months to 19 years (10.91 ± 4.99 years; Table 1). The demographic characteristics, genetic abnormalities, gonadal characteristics, surgical procedures, and occurrence of gonadal tumors are summarized in Tables 1, 2. Among the 22 studied patients, 11 presented with short stature, six with amenorrhea and delayed puberty, two with bilateral inguinal hernia, and one with a massive abdominal mass, whereas two remained asymptomatic, namely, one with a 17α-hydroxylase/17,20-lyase deficiency (17OHD) family history and one with chromosomal abnormalities during healthy screening. Parental consanguinity and abnormal medication during pregnancy were denied in all families. A 10-year-old girl (Patient No. 1) reported a family history of 17OHD. Her 46,XY elder sister presented with amenorrhea and infertility and underwent bilateral gonadectomy after the diagnosis of 17OHD in another hospital (no genetic data details). Another 2-year-old patient (Patient No. 7) exhibited congenital heart disease with total anomalous pulmonary venous drainage (TAPVD). Patients were divided into three groups according to chromosomal analysis, gonadal characteristics, and genetic abnormalities (Table 3). A total of 10 patients were diagnosed with Turner syndrome after presented with short stature and having mosaic karyotypes with Y chromosome, bilateral streak gonads, an infantile uterus, and normal external genitalia. Five patients were identified with androgen synthesis/action disorders, including two patients with 17OHD, two patients with complete androgen insensitivity syndrome (CAIS), and one patient with steroid 5α-reductase 2 deficiency. Another patient with short stature and bilateral testes carried a *NR5A1* gene mutation. For the remaining six patients with the 46,XY karyotype, 46,XY CGD was diagnosed. Streak gonads were identified in 16 (72.7%) patients based on the clinicopathological analyses.

**TABLE 3:** Presenting clinical features and number of gonadal tumors by etiology.

	ETIOL 1^a^	ETIOL 2^b^	ETIOL 3^c^	Total
**Presenting symptoms**				
Short stature	9	1	1	11
Amenorrhea	1	3	3	7
Inguinal hernia/mass	Nil	Nil	2	2
Abdominal mass	Nil	1	Nil	1
Other	Nil	1	Nil	1
Total	10	6	6	22
Morphology	Streak gonads	Streak gonads	Testis	
**Type of gonadal tumor**				
Dysgerminoma	Nil	2	0	2
Gonadoblastoma	1	2	0	3
Leydig cell tumor	Nil	Nil	1	1
Total	1/10	4/6	1/6	6/22

^a^ETIOL 1: TS SRY+. ^b^ETIOL 2: 46,XY complete gonadal dysgenesis (46,XY CGD or Swyer syndrome). ^c^ETIOL 3: 46,XY androgen synthesis/action disorder. ETIOL, etiology.

All cases in this study received Sanger sequencing of the *SRY* gene, and no mutations were detected. The parents of Patients No. 3, 4, and 5 chose WES to detect the potential pathogenic mutations after consultation, although Sanger sequencing of the *AR* and *SRD5A2* genes was recommended first according to our comprehensive gene detection strategy. Genetic analysis by whole-exome sequencing revealed that there were two 46,XY DSD patients harboring mutations of c.1226C > G, c.707T > G, and c.985_987delTACinsAA in the *CYP17A1* gene, which had been reported before (11–13). A novel *NR5A1* mutation c.244 + 1G > C (splicing) was identified and predicted to be pathogenic by bioinformatics tools in one 46,XY DSD (Patient No. 6) presented with short stature. The patient’s external genitalia showed a female phenotype with mild clitoromegaly. The vaginal and urethral openings were observed at the perineum. Laparoscopic exploration found bilateral gonads were located in the pelvic cavity, and histology of the gonadal biopsy showed bilateral dysplastic testicular tissue without tumor. Furthermore, we reported four other novel disease-associated mutations in our cohort of 46,XY DSD by whole-exome sequencing: c.1314_1315del, c.2245G > A in the *AR* gene and c.419_421delinsATTC, c.547-1G > C in the *SRD5A2* gene. All were predicted as pathogenic by bioinformatics tools. Thus, considering the typical clinical manifestations of our patients and WES did not reveal other suspected pathogenic mutations, we believed that these novel mutations were the etiology of 46,XY DSD. *In vitro* functional studies are needed to further investigate the potential biological function of the *de novo* variations.

**FIGURE 2:**
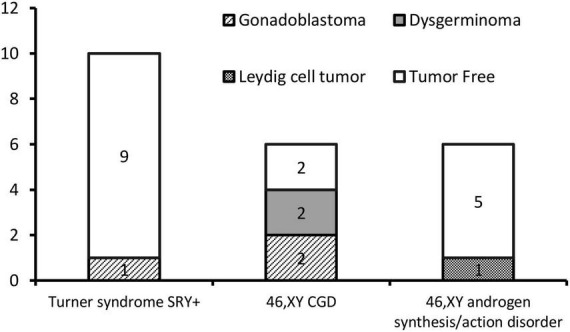
Number of gonadal tumors according to different etiologies.

The gonadal neoplasms were presented as a large abdominal tumor and an irregular mass on the streak gonad in two cases with dysgerminoma (Patients No. 8, 9), while the remaining four cases were presented as microscopic neoplasms on the surgical pathology (Table 2). In our series, apart from one 46,XY 17OHD patient with bilateral Leydig cell tumor who had dysplastic testicular gonads, all the gonadal tissues with tumor were of streak pattern. The definitive diagnosis of GCNIS/GB, dysgerminoma, or other gonadal neoplasms was confirmed with histopathological features and immunohistochemical stains of markers like PLAP, SALL4, OCT3/4, TSPY, and FOXL2. The percentage of patients with gonadal tumors was calculated to assess the risk of gonadal tumor and malignancy according to the three etiologies. The postoperative pathological examination identified gonadal tumors in six patients; 3, 2, and 1 patients had, gonadoblastoma, dysgerminoma, and Leydig cell tumor, respectively. The overall rate of gonadal tumors was 27.3% (6/22). 46,XY CGD had the highest rate at 66.7% (4/6 patients). One of the six patients with gonadal tumor (16.6%) was classified among 46,XY androgen synthesis/action disorders. One of the 10 patients with TS and a Y chromosome had bilateral gonadoblastoma (10%; Figure 2). The youngest patient with a tumor was a 2-year-old girl with 46,XY CGD who was diagnosed with bilateral gonadoblastoma (Patient No. 7). She also had congenital heart disease of TAPVD and received surgery in our hospital at the age of 5 months.

All patients underwent laparoscopic bilateral gonadectomy or gonadal biopsy, except for one patient with a massive abdominal tumor who received radical tumor resection after preoperative chemotherapy. In this exceptional case (Patient No. 9), the presence of dysgerminoma determined from postoperative gonadal pathology initiated further detection of the 46,XY karyotype in peripheral blood lymphocytes, conflicting with the female phenotype. The 46,XY CGD was diagnosed after a complete assessment of the patient’s clinical condition. After consultation with the parents, the remaining left gonad was surgically removed after the first operation. Pathological assessment of the resected gonad confirmed a streak gonad free of tumor tissue. A 14-year-old patient with 46,XY CGD (Patient No. 8) was found with dysgerminoma intraoperatively; a laparoscopic bilateral gonadectomy was performed. Preoperative ultrasonography revealed an enlarged left ovary, 1.4 cm × 2.3 cm × 0.8 cm, and postoperative histopathology confirmed bilateral dysgerminoma. Imaging studies showed no metastasis in the two patients with dysgerminoma. The postoperative course was uneventful in both patients. For each of the patients with dysgerminoma, a chemotherapeutic combination of cisplatin, etoposide, and bleomycin (PEB) was applied. After surgery, each patient was followed up by a multidisciplinary team, comprising a urologist, an endocrinologist, and a psychologist. Further hormone replacement therapy was administered at the appropriate age.

## Discussion

With the wide spectrum of phenotypes, ranging from variable degrees of external genitalia undervirilization to normal female external genitalia, the diagnosis of the unambiguous phenotypic female patient with DSD and Y-chromosome material is often delayed until puberty. These patients often present with short stature or amenorrhea. The presence of the Y chromosome may increase the risk of developing gonadal tumors in these patients (14–20). However, gonadal tumors in patients with gonadal dysgenesis are frequently found at a young age (21, 22). Our study retrospectively analyzed the risk of gonadal tumor of different etiologies in phenotypic pediatric and adolescent female patients with DSD and reconfirmed the broad applicability of the stratified strategy principle in the clinical management of gonadal tumor risk, including the pediatric population (1, 23, 24). Pediatric patients with 46,XY CGD in this study had a significantly increased risk of gonadal tumors and underwent prophylactic gonadectomy immediately after the diagnosis was confirmed, whereas those with Turner syndrome with Y chromosome and 46,XY androgen synthesis/action disorders had a relatively lower risk.

The currently recognized risk factors for developing a malignant gonadal tumor in DSD mainly include the presence of the GBY region of the Y chromosome. Additional risk factors include dysplastic gonads with embryonic germ cell markers, such as OCT3/4 (octamer binding transcription factor 3/4), and abnormal gonadal location (1, 25). The early diagnosis of these rare tumors requires the identification of precursor lesions, such as GCNIS in the testis and gonadoblastoma in the ovary/dysgenetic gonad (26). If left untreated, most cases of precursor lesions progress to an invasive malignant type-II germ cell tumor/germ cell cancer (GCT/GCC). Phenotypes may appear comparable in unambiguous phenotypic female patients with DSD and Y-chromosome material, but their underlying causes and pathogenesis are diverse, complicating an accurate diagnosis. For DSD of bilateral gonadal dysplasia, gonadotropin levels are usually high, which may aid in a differential diagnosis (27). The diagnosis of some rare single-gene diseases, such as 17OHD, caused by *CYP17A1* gene mutation, often depends on molecular biological detection methods, such as Sanger sequencing or whole-exome sequencing.

The 46,XY CGD was one of the three etiologies with the highest proportion of gonadal tumors in this study due to the high incidence of gonadal tumors in bilateral streak gonads. In this study, 16 of the 22 patients had streak gonads, and 5 (31.2%) of these patients had gonadal tumors. Among the five cases of gonadal tumors, two of them were malignant dysgerminomas, which required surgical intervention and adjuvant chemotherapy. Premalignant lesions of the GCT in this kind of DSD can occur at an early age of 15 months (28). In this case series, the youngest patient diagnosed with a tumor was a 2-year-old patient (Patient No. 7) with 46,XY CGD who was diagnosed with bilateral gonadoblastoma. Gonadoblastoma is the precursor lesion of GCC, and it is the most common tumor associated with 46,XY CGD (29, 30). The presence of the Y chromosome is necessary for the development of gonadoblastoma in a dysgenetic gonad. Although gonadoblastoma is benign, malignant transformation to seminomatous or non-seminomatous tumors can occur over years. In this study, gonadal tumors were identified in 66.7% (4/6) of the individuals with 46,XY CGD, and 50% (2/4) of these tumors were malignant dysgerminomas that needed surgical intervention and adjuvant chemotherapy. Due to the higher risk of gonadal tumors in 46,XY CGD, prophylactic bilateral gonadectomy is strongly recommended as soon as the diagnosis is confirmed.

Recently, a retrospective study by the Children’s Oncology Group compared the event-free and overall survival rates of patients with malignant ovarian GCTs in the context of gonadal dysgenesis or normal gonadal development. Patients with malignant GCTs with gonadal dysgenesis were concluded to have a worse prognosis than those with normal gonads. These findings supported the recommendation for early bilateral gonadectomy in patients with any ovarian GCT, with a contralateral streak ovary or gonadoblastoma after histological assessment (31). The high incidence of gonadal tumors in this study was consistent with the findings of most previous literature reports. Considering the poor prognosis of those with malignant ovarian GCTs with dysgenetic gonads, prophylactic bilateral gonadectomy is the commonly recommended optimal treatment for these particular patients with DSD, even at a young age.

Turner syndrome is a special type of gonadal dysgenesis that affects 1 in 2,000 live births (32). Women with mos 45,X/46,XY and Turner syndrome may develop gonadoblastoma, determined by the presence of the GBY region in their karyotype. The risk of developing gonadoblastoma among individuals with SRY-positive Turner syndrome remains controversial, according to the published literature. Generally, in clinical practice, SRY-positive Turner syndrome is in the moderate-risk group of gonadal tumors, and prophylactic bilateral gonadectomy is recommended. However, in Denmark, the occurrence of gonadoblastoma among Y-positive patients seemed to be low (7–10%) (5, 33). Conversely, in a Japanese series of pediatric patients with DSD with bilateral streak gonads, four of seven patients with Turner syndrome who had a Y-mosaic karyotype and an unambiguous female phenotype were diagnosed with gonadoblastoma (4/7, 57.1%). This increased risk of gonadoblastoma seems present in younger age groups but not in adults (8). The differences in results between these studies may be due to the small number of participants, ethnic differences, or research bias. A large national cohort study in the United Kingdom on 211 women with Turner syndrome who were Y-chromosome-positive and aged between 18 months and 22 years at tumor diagnosis described five cases of ovarian gonadoblastoma that occurred during follow-up. The cumulative risk of gonadoblastoma by the age of 25 years in Y-chromosome-positive women was 7.9% (95% CI: 3.1–19.0) (34). A high level of discrepancy among patients with Turner syndrome and the presence of the Y chromosome highlights the difficulty in providing precise guidelines for such patients.

In this study, in SRY-positive girls with Turner syndrome without signs of virilization, the rate of tumor occurrence was relatively low (1/10, 10%), which is consistent with findings in most of the published literature. Therefore, in cases where the girls or parents are opposed to prophylactic bilateral gonadectomy or if surgery is contraindicated, gonadectomy can be postponed or the patients should be followed-up. Although currently no diagnostic tools that allow reliable monitoring of abdominal gonads for the presence of GCNIS/GB are available, new and promising micro RNA- and SNP-based screening tests, such as the miR-371a-3p test, are underway (35). Due to the high reported incidence of gonadoblastoma, great attention must be paid to this group of patients with DSD. However, spontaneous thelarche and spontaneous menarche have been reported in girls with Turner syndrome with Y-chromosome material (36). In this study, the fibrous tissue rather than germ cells was found in the removed gonadal tissues, indicating that, in most cases, the retention of non-functional streak gonads was not clinically significant. The risk of gonadal tumors, however, was relatively low in girls with Turner syndrome with mos 45,X/46,XY, as revealed by this study and most of the published reports, which recommend taking the surgical decision after discussing the tumor risk and gonadal function potential.

Regarding phenotypic female patients with DSD caused by testosterone biosynthesis or androgen action disorders, as in CAIS, most DSD experts support conservative management of the gonads because the risk of gonadal tumor during childhood is low. Although gonads with testosterone biosynthetic defects are generally assumed not to be prone to neoplasia in prepubertal or pubertal patients, sporadic carcinoma *in situ* has been reported in the non-dysgenetic testis in phenotypic female adolescents (37, 38). Therefore, early identification and timely treatment of such patients is clinically important. Furthermore, according to the recent follow-up data of a large number of cases in China, adult patients with CAIS may exhibit gonadal tumors that could be malignant (18). King et al. (39) found no association between age at gonadectomy and bone mineral density (BMD) and no reduction in BMD after gonadectomy in participants with CAIS. Therefore, postpubertal or adult gonadal resection is a relatively safe option. Pediatric patients with CAIS who refuse gonadectomy, despite the increasing tumor risk in adulthood, should be closely followed-up with long-term regular ultrasound examination and detectable biomarkers, taking into consideration the low specificity of imaging [ultrasound scan (US), MRI], and lack of reliable biomarkers for early detection of emerging tumors in the abdominal gonads.

Patients with 46,XY DSD with variants in the *NR5A1* gene commonly cause a wide range of phenotypes. In this study, a rare phenotypic female patient with a novel *NR5A1* mutation c.244 + 1G > C (splicing) and bilateral dysplastic testes was identified. Our patient (Patient No. 6) is very similar to a previously reported case with a c.244G > T mutation in the *NR5A1* gene by Yu et al. (40). At birth, the patient’s external genitalia showed a female phenotype, and at the age of 12 years, she was referred to the hospital because of clitoris hypertrophy and primary amenorrhea. No Müllerian structure was found, and bilateral gonads were located in the pelvic cavity. Histology of the left and right gonad revealed dysplastic testicular tissue after laparoscopic bilateral gonadectomy. Another patient of steroid 5α-reductase 2 deficiency presented with bilateral inguinal hernia and complete female external genitalia without signs of virilization in our cohort, although hypospadias was well recognized as the main malformation in these cases. In these rare phenotypic female cases, if the child was ultimately raised as female gender, gonadectomy would be postponed until an appropriate age due to the low gonadal tumor risk.

In female patients with 46,XY 17OHD DSD, the data on gonadal tumor risk regarding testicular adrenal rest tumors (TART), Leydig cell hyperplasia, and gonadal malignancy is limited. Some data on gonadectomy was available for 62 patients in a review, and none reported TART or malignancy (41). A recent study reported two female patients with 46,XY 17OHD who were diagnosed with a testicular malignancy in a pathological study after laparoscopic orchiectomy (42). In this study, one patient with 46,XY 17OHD was confirmed to have bilateral Leydig cell tumor, a gonadal neoplasm derived from the gonadal stroma not belonging to GCTs. Leydig cell tumors may cause endocrine changes leading to virilizing syndromes due to increased production of androgen. However, in our case, no virilizing phenotype was present, perhaps due to the inhibition of androgen synthesis caused by genetic mutations. While the majority of these tumors follow a benign clinical course, 10% of the tumors are malignant (43). Our study provided data on the gonadal tumor risk of this rare type of DSD, showing that despite the low gonadal tumor risk of patients with DSD associated with testosterone biosynthetic defects, a monitoring approach with close follow-up is bothersome because of the low specificity of imaging (US and MRI) and lack of biomarkers for detecting emerging tumors in the abdominal gonads. Discussing gonadectomy with patients and parents at an appropriate age is an appropriate and reliable treatment option.

## Conclusion

This study assessed the risk of gonadal tumors according to different etiologies in Chinese pediatric and adolescent patients with DSD with an unambiguous female phenotype and Y chromosome in a single center. Among patients in this study, the proportion of streak gonads with gonadal tumors was concerning, especially in patients with 46,XY CGD. Our findings support literature that recommends bilateral prophylactic gonadectomy, even in childhood, for patients with 46,XY CGD. For girls with SRY-positive Turner syndrome, the rate of tumor occurrence was relatively low, and for those who refuse prophylactic gonadectomy, surgery can be postponed or patients should be followed up closely. Due to the abnormal anatomical position of the gonads and their difficulty in monitoring, more attention should be paid to the 46,XY DSD children and adolescents with androgen synthesis/action disorders who choose to preserve the gonads, and it is necessary to identify new biomarkers to detect the occasional gonadal neoplasm during adolescence, such as the case in our study. In view of the limited number of patients in this study, a more rigorous multicenter study with a longer follow-up duration is needed to support these conclusions.

## Data availability statement

The original contributions presented in this study are included in the article/supplementary material, further inquiries can be directed to the corresponding author.

## Author contributions

LL conceptualized, designed, and conducted the study, participated in the acquisition of clinical data, wrote the initial draft of the manuscript, and agreed to be responsible for all aspects of the work to ensure accuracy and integrity. FL participated and guided the clinical hormone replacement therapy treatment of patients with DSD and participated in the revision of the final manuscript. XW reviewed the manuscript critically and approved the final version of the manuscript for publication. All authors were involved in revising the original draft of the manuscript, approved the final manuscript as submitted, and agreed to be accountable for all aspects of the work.

## Conflict of interest

The authors declare that the research was conducted in the absence of any commercial or financial relationships that could be construed as a potential conflict of interest.

## Publisher’s note

All claims expressed in this article are solely those of the authors and do not necessarily represent those of their affiliated organizations, or those of the publisher, the editors and the reviewers. Any product that may be evaluated in this article, or claim that may be made by its manufacturer, is not guaranteed or endorsed by the publisher.

## Abbreviations

17OHD: 17α-hydroxylase/17,20-lyase deficiency
CAIS: complete androgen insensitivity syndrome
DSD: disorders of sex development
GCT: germ cell tumor
CGD: Complete Gonadal Dysgenesis
TS: Turner syndrome.
